# Sick-Headache Cured with One Dose of Lachesis^cm^

**Published:** 1892-02

**Authors:** Edward Rushmore

**Affiliations:** Plainfield, N. J.


### SICK-HEADACHE CURED WITH ONE DOSE OF LACHESIS$^{CM}$.

Edward Rushmore, M. D., Plainfield, N. J.

Six months ago, Mrs. —— called on me to say that she was a great sufferer from headache, which always began with dim and aching eyes. It is in the temples and eyes, worse at the right side, a sharp neuralgic pain. If she cannot be still with it she has nausea and very bitter vomiting. Sometimes she cannot lie still a minute, at others she cannot stir. It is brought on by the least fatigue. Keeps her in bed all day, and she hardly gets over one attack before another comes. Mental excitement, like receiving a call, gives her headache. She is very cold with the headaches, and has a very bitter mouth with and after them. She wants to close the eyes with headache, which is relieved by sleep. Smarting in eyeballs and dimness of sight for several days after the headache. Had a terrible time with her heart lately, during the headache; "skipping beats," and soreness about the heart and pain in the side after the headache. Loss of appetite after the headache. Menses regular, painless, too free. Leucorrhœa many years. I gave her one dose of Lachesis$^{cm}$, Fincke, dry on the tongue. A week later she reported that the headache she had on first visiting me left her as she went from my house, but she has had a constant light ache in the head ; the head heavy in the morning, but no severe attack. The heart better. No medicine. That was the last I saw of her for nearly six months. When being called to see her for other symptoms, she told me she had wished to come and tell me that what I gave her had completely cured her headaches.

